# Diagnosis and management of sigmoid leiomyosarcoma presenting with intussusception: a case report

**DOI:** 10.1093/jscr/rjae795

**Published:** 2024-12-19

**Authors:** Sami A El-Boghdadly, Alaa M Alshareef, Abdulrahman K Almisfer, Ibrahim M Momen

**Affiliations:** Department of General Surgery, King Abdulaziz Medical City, Ministry of National Guard Health Affairs, Ar Rimayah, Riyadh 14611, Saudi Arabia; Department of General Surgery, Dr Sulaiman Al habib Medical Group, Khurais Br Rd, Ar Rayyan, Riyadh 14212, Saudi Arabia; Department of General Surgery, King Abdulaziz Medical City, Ministry of National Guard Health Affairs, Ar Rimayah, Riyadh 14611, Saudi Arabia; College of Medicine, King Saud Bin Abdulaziz University for Health Sciences, Ar Rimayah, Riyadh 11481, Saudi Arabia

**Keywords:** leiomyosarcoma, intussusception, sigmoid colon, colon cancer, case report

## Abstract

Sigmoid leiomyosarcoma is a rare type of cancer that originates from smooth muscles of the sigmoid colon. This case report details a 50-year-old female found to have this condition, presenting to the emergency department with intussusception. A CT scan revealed two intraluminal masses, with the largest 10 cm from the anal verge. A colonoscopy and biopsy revealed a large obstructing mass with atypical spindle-shaped cells. This case shows a rare type of cancer presenting in a rare complication and highlights the approach done in the multi-disciplinary tumor board meeting involving gastroenterologists, radiologists, surgeons, and oncologists, leading to prompt diagnosis and surgical resection.

## Introduction

Leiomyosarcoma (LMS) is a malignant neoplasm that accounts for 20% of all sarcomas [1]. It originally arises from smooth muscles and is typically characterized by spindle-shaped cells and associated with RB1 and PTEN tumor suppressor gene mutations [2]. This type of cancer occurs mostly in the retroperitoneum, uterus, extremities, and trunk. LMS is extremely rare to originate in the colon with only <1% of all smooth muscle tumors in the gastrointestinal tract [1, 3, 4]. When colon leiomyosarcoma presents with intussusception, it poses unique challenges in terms of diagnosis and management. Here, we show a case of LMS presenting as two large sigmoid polyps causing sigmoid-rectal intussusception.

## Case presentation

A 50-year-old Saudi female with a medical history of gastroesophageal reflux, hypothyroidism, endometrial carcinoma, and ovarian serous adenocarcinoma presented to the emergency department complaining of 1 month history of intermittent severe lower abdominal pain. This pain was associated with constipation, rectal bleeding, and unintentional weight loss from 88 to 55 kg. Physical examination showed a palpable mass in the left lower quadrant of the abdomen with mild tenderness.

**Figure 1:**
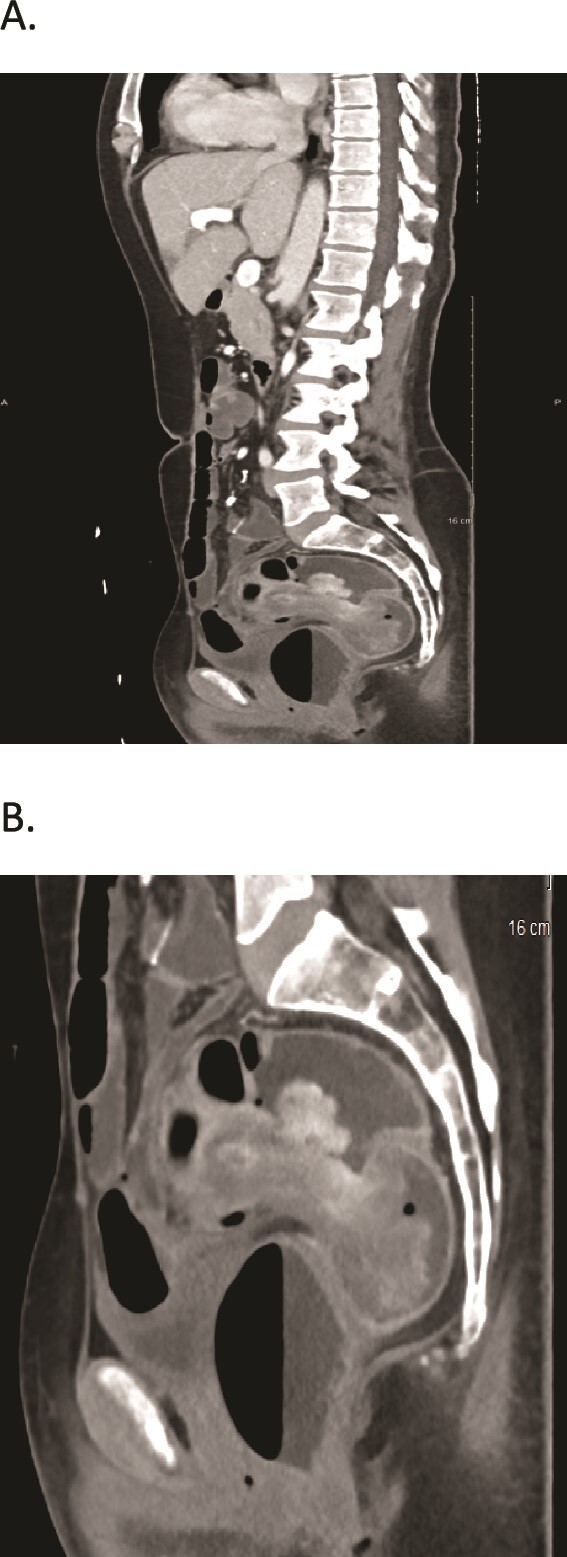
(A, B) CT scans show two large fungating intraluminal sigmoid masses. The largest is 5 × 5.5 × 4.3 (AP, transverse), about 10 cm from the anal verge, and associated with “target sign” in the sigmoidorectal area.

A computed tomography (CT) scan was done and it revealed two large fungating intraluminal sigmoid masses. The largest is 5 × 5.5 × 4.3 (AP, transverse), about 10 cm from the anal verge, and associated with “target sign” in the sigmoido-rectal area suggestive of intussusception with no signs of bowel obstruction or lymph node involvement (Fig. 1).

**Figure 2:**
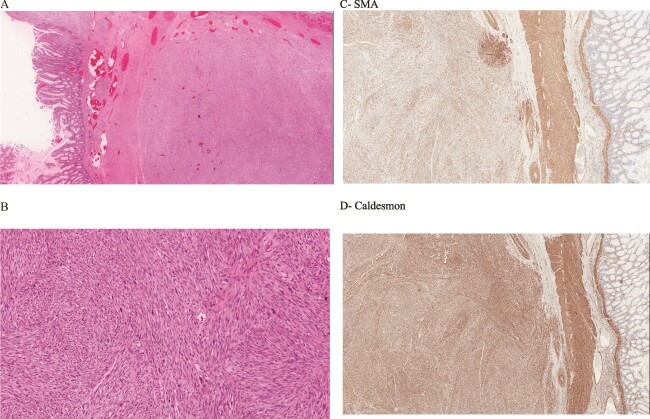
(A, B) Histology image shows spindle-shaped cells with plump, blunt-ended nuclei and moderate to abundant, pale to brightly eosinophilic fibrillary cytoplasm. The cells are arranged in long intersecting fascicles with abundant mitosis. The nuclei exhibit moderate nuclear pleomorphism. (C) Positive for SMA stain. (D) Caldesmon.

Following the CT scan, the patient underwent a colonoscopy with biopsy. The endoscopic examination revealed a large, friable mass partially obstructing the sigmoid colon, resulting in intussusception. Multiple biopsies were obtained, and histopathological analysis revealed spindle-shaped cells with plump, blunt-ended nuclei and moderate to abundant, pale to brightly eosinophilic fibrillary cytoplasm. The cells are arranged in long intersecting fascicles with abundant mitosis. The nuclei exhibit moderate nuclear pleomorphism (Fig. 2). Immunohistochemistry stains were found positive for SMA and Caldesmon and negative for CD117 (C-KIT). These findings confirmed the diagnosis of sigmoid leiomyosarcoma.

Given the patient's diagnosis of sigmoid leiomyosarcoma presenting with intussusception, the case was discussed in multi-disciplinary tumor board meeting. Surgical excision was deemed necessary to address both the intussusception and the malignancy. The patient underwent exploratory laparotomy during which a segmental resection of the sigmoid colon with primary anastomosis, and lymph node dissection was performed.

The patient had an uneventful postoperative recovery and was discharged on postoperative day 6. Histopathological examination of the resected specimen revealed a 7-cm leiomyosarcoma with clear surgical margins and no lymph node involvement. The tumor was classified as stage II (T1, N0, Mx) according to the American Joint Committee on Cancer (AJCC) staging system 8th edition [5]. Postoperative histopathological findings were discussed in multi-disciplinary tumor board meeting. Consensus was to start the patient on adjuvant chemotherapy. The results of the meeting were discussed with the patient, and she agreed to undergo six cycles of doxorubicin-based chemotherapy to reduce the risk of recurrence.

Regular follow-up visits were scheduled for the patient after completing chemotherapy by physical examinations, imaging studies (CT scans), and tumor markers assessments every 3 months during the first year, followed by 6-month intervals thereafter. After completing her first year follow-ups, the patient remained asymptomatic, imaging studies showed no evidence of tumor recurrence or metastasis, and her tumor markers remained within normal ranges.

## Discussion

Commonly, GI tract LMS originates in stomach and small bowel and presents with abdominal pain, melena, and weight loss [6, 7]. On the other hand, colon LMS’s presentations are unclear due to the rarity of this type of cancer and lack of case reports. In previously reported cases of colon LMS, symptoms like constipation and lower abdominal pain were reported, while another case report showed hematochezia and change in bowel habits only [8, 9].

Furthermore, our case did not only have sigmoid LMS but also developed sigmoido-rectal intussusception, which is rare complication present in adults [10]. Diagnosing sigmoid leiomyosarcoma complicated by recto-sigmoidal intussusception requires a comprehensive approach involving imaging studies, endoscopic examinations, and histopathological analysis.

CT is the best imaging modality for intussusception in adults. It typically shows “target sign” that suggests intussusception and plays a role in establishing biopsy targets for further evaluation. [9, 11]. In our case, masses seen on CT scan were the primary cause of intussusception which prompted an urgent colonoscopy.

Histopathological analysis of the masses revealed typical findings of classic leiomyosarcoma, which showed spindle-shaped cells arranged in intersecting fascicles with elongated and hyperchromatic nuclei with eosinophilic cytoplasm [2]. It is crucial to distinguish LMS from gastrointestinal stromal tumors (GISTs) as they are the most common mesenchymal tumors in the GI tract where the only different histological feature between them is that GISTs are positive for CD117 and CD34 while LMSs are not [9, 12].

Surgical intervention remains the cornerstone of treatment for sigmoid leiomyosarcoma presenting with intussusception. The extent of surgical resection should be carefully determined based on tumor size, location, and staging. In our case, the patient underwent exploratory laparotomy with segmental resection of the sigmoid colon and primary anastomosis [2].

Staging sigmoid leiomyosarcoma is vital for determining the need for additional therapy and predicting outcomes. The AJCC staging system is commonly used [5]. In our case, the tumor was stage II, indicating local involvement without lymph node metastasis. Adjuvant chemotherapy, often recommended for high-grade, large tumors, or lymph node involvement, was indicated for our patient due to her history of multiple malignancies. She completed six cycles of doxorubicin-based chemotherapy with no significant side effects.

Postoperative surveillance, including exams, imaging, colonoscopies, and tumor marker assessments, is crucial for detecting recurrence or metastasis. Our patient showed a good response with no recurrence or metastasis at her first year follow-ups.

While our case report demonstrates successful diagnosis and management, limitations exist. The rarity of sigmoid leiomyosarcoma with intussusception limits large-scale studies, hindering the establishment of standardized guidelines. Additionally, the impact of adjuvant therapy on long-term outcomes remains unclear, requiring further research to assess its effectiveness.

## Conclusion

In conclusion, we report a case of sigmoid colon leiomyosarcoma with intussusception, successfully treated with open segmental resection and primary anastomosis, with no postoperative complications. Due to its rarity, diagnosing and managing sigmoid LMS with intussusception are challenging. A multi-disciplinary tumor board meeting approach is vital for accurate diagnosis, intervention, and consideration of adjuvant therapy. Long-term surveillance is necessary to detect recurrence or metastasis. Further research is needed to establish guidelines for better diagnosis and management of this rare condition, improving patient outcomes.
